# Dietary cholesterol intake and egg consumption in relation to all-cause and cardiovascular mortality after stroke

**DOI:** 10.1038/s41598-025-19028-0

**Published:** 2025-10-08

**Authors:** Lingfan Xia, Tong Xu, Zhenxiang Zhan

**Affiliations:** 1 Department of Neurology, Taizhou First People’s Hospital, Taizhou, 318000 Zhejiang Province China; 2 Department of Neurology, The Second Affiliated Hospital and Yuying, Children’s Hospital of Wenzhou Medical University, Wenzhou, China; 3 Department of Neurology, Affiliated Jinhua Hospital, Zhejiang University School of Medicine, Jinhua, China

**Keywords:** Cardiovascular disease, Cholesterol, Diet, Egg, Mortality, Stroke, Nutrition, Stroke

## Abstract

The findings on the associations of dietary cholesterol intake and egg consumption with mortality in the general population remain inconclusive. Our objective was to evaluate all-cause and cardiovascular mortality in relation to dietary cholesterol intake and egg consumption in a cohort of stroke survivors, a particularly vulnerable population. We conducted a prospective cohort analysis of 1367 stroke survivors recruited across the United States between 1999 and 2018. Dietary information was evaluated by a 24-h dietary recall at baseline obtained from NHANES while mortality data were derived from the National Death Index with follow-up through 2019. To investigate the associations of dietary cholesterol intake or egg consumption with all-cause and cardiovascular mortality, Cox proportional hazards and restricted cubic splines models were utilized. After a median follow-up of 79.0 months, the incidence per 1000 person-years was 59.9 (95% confidence interval [CI], 55.2–64.9) for all-cause and 23.3 (95% CI, 20.4–26.5) for cardiovascular mortality. Each 100 mg/1000 kcal/day elevation in cholesterol intake was related to raised risk of all-cause (hazard ratio [HR], 1.16; 95% CI, 1.05–1.27) and cardiovascular mortality (HR, 1.15; 95% CI, 1.00–1.31). A greater risk of all-cause mortality was shown in participants consuming > 1 egg/day (HR, 1.40; 95% CI, 1.06–1.84). The dose-response analysis highlighted the lowest risk of all-cause mortality when egg consumption was around 33.3 g/day. The observed correlations of dietary cholesterol intake or egg consumption with all-cause and cardiovascular mortality were stronger in the subgroups with cardiometabolic diseases (obesity, hypertension, diabetes mellitus, hyperlipidemia) or risk factors for cardiometabolic diseases (advanced age). Among stoke survivors, greater dietary cholesterol connects to an escalated risk of all-cause and cardiovascular mortality in a linear manner. Moderate egg consumption (≤ 1 egg/day) is relatively safe and excessive consumption links to an elevated risk of all-cause mortality.

**Supplementary Information:**

The online version contains supplementary material available at 10.1038/s41598-025-19028-0.

## Introduction

Stroke remains a global Health problem, affecting approximately 142 per 100, 000 persons per year^1^. Recent reports uncovered that stroke represents the second leading cause of mortality and the third leading cause of disability among non-communicable disorders worldwide^2^. By 2021, the number of stroke survivors has reached 93.8 million and will keep growing in the future^2^. This highlights the need for interventional strategies to modify health status and longevity in patients after stroke.

Cholesterol is essential for maintaining cellular membrane structure and critical signal transduction and is involved in important physiological functions comprising intestinal absorption of lipids, glucose metabolism, reproductive function, and bone homeostasis^3^. However, excess cholesterol accumulation in the artery wall has cytotoxic activity and leads to the formation of atherosclerotic plaques, endothelial dysfunction, and chronic inflammation^4^. A well-regulated cholesterol metabolism is crucial for the body’s normal functioning, and impaired cholesterol homeostasis is involved in various systemic disease development, including cardiovascular, tumors, Alzheimer’s, Parkinson’s, and immune system diseases^5^. Eggs are a primary source of dietary cholesterol, with around 186 mg of cholesterol in a large boiled egg (around 50 g)^6^. In addition, eggs contain a wide range of other high-quality nutrients, including protein, fatty acids, minerals, and vitamins (except for vitamin C)^6^. As a common and affordable food, egg consumption is pretty universal among the population.

The recent meta-analyses of cohort studies did not find significantly increased risk of coronary heart disease and stroke in people with higher dietary cholesterol intake or egg consumption^7,8^. Owing to the inadequate evidence, the 2015–2020 Dietary Guidelines for Americans eliminated the limit of dietary cholesterol intake of 300 mg/day and proposed that “individuals should eat as little dietary cholesterol as possible while consuming a healthy eating pattern” and “a healthy eating pattern includes egg consumption”^9^. The associations of dietary cholesterol intake and egg consumption with mortality continue to be contentious despite extensive research over several decades, with divergent findings including positive^10–13^, negative^14,15^, and null associations^16,17^. Two newly published meta-analyses have made a detailed summary of previous studies, but still drew ambiguous conclusions^18,19^. Incompatible results between conventional and dose-response meta-analyses, high heterogeneities across the analyzed studies, and low-level certainty of evidence are the inherent defects of these meta-analyses. As some studies reported nonlinear relationships between dietary cholesterol intake and egg consumption and mortality risk^20–22^, while some studies did not conduct nonlinear relationship detection, we speculated that potential nonlinear relationships may lead to the divergent findings in previous work. In addition, the divergent findings may be due to the various composition of study populations (e.g., age, sex, and race). Although the effects of consumption of dietary cholesterol and eggs on mortality have been intensively studied in the general population, evidence is scarce among population with stroke. As a critical cardiovascular endpoint event, stroke has dyslipidemia as its primary modifiable risk factor^23^. Clinical practice guidelines highlight the importance of lipid control in stroke patients, with specific targets requiring low-density lipoprotein cholesterol levels below 70 mg/dL^24^. As an exogenous source of cholesterol, dietary cholesterol intake and egg consumption require particular attention in secondary prevention strategies for stroke survivors.

Therefore, our aim was to explore the associations of dietary cholesterol intake and egg consumption with all-cause and cardiovascular mortality in a nationally representative cohort of stroke survivors. As previous work conducted in the general population reported inconsistent results, we implemented nonlinear relationship detection and subgroup analyses to accurately depict the associations of consumption of dietary cholesterol and eggs with mortality as much as possible.

## Methods

### Study sample

The participants of this study were screened from the National Health and Nutrition Examination Survey (NHANES), whose goal is to evaluate the health and nutritional status of residents in the United States (US). In a two-year cycle, NHANES selects approximately 10,000 people to construct a nationally representative sample by complex, multistage probability sampling. Combining interviews, physical examinations, and laboratory tests, NHANES provides detailed sociodemographic, dietary, and health-related statistics. This study was approved by the National Center for Health Statistics (NCHS) Ethics Review Board and conducted in accordance with the Declaration of Helsinki. All participants signed the informed consent. We followed the Strengthening the Reporting of Observational Studies in Epidemiology (STROBE) reporting guideline.

**Fig. 1:**
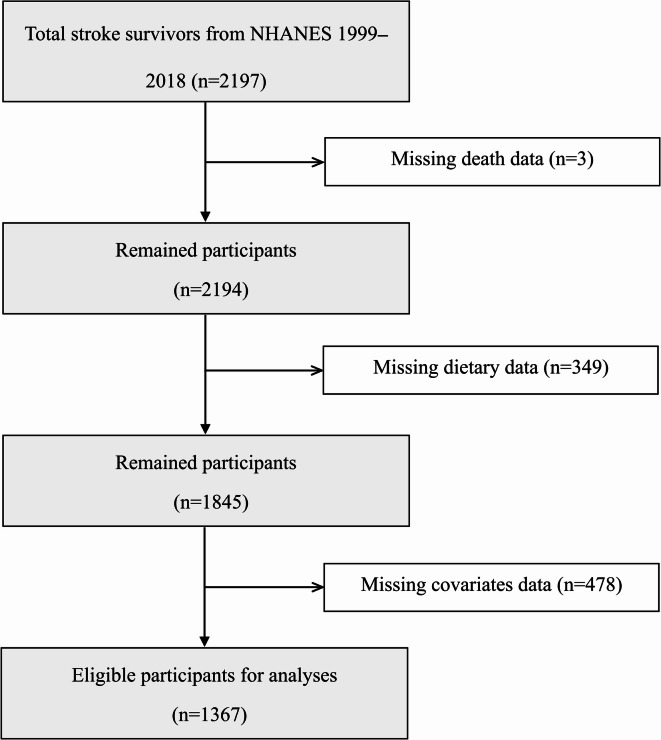
Flow diagram of selecting included participants.

Participants aged ≥ 20 years were interviewed to report their Health conditions in ten cycles of NHANES, 1999–2000 to 2017–2018. Individuals who responded affirmatively to the question, “Has a doctor or other health professional ever told you that you had a stroke?“, were classified as stroke survivors. The question was asked by qualified interviewers utilizing the Computer-Assisted Personal Interview (CAPI) system, which incorporates integrated consistency checks to minimize entry inaccuracies and employs online help screens to aid interviewers in defining substantial terms within the questionnaire. Upon the documentation of unusual, inconsistent, or unrealistic responses, the interviewer was promptly alerted and directed to ascertain or modify the first response. Figure 1 illustrates the study sample selection procedures. We initially identified a total of 2197 stroke survivors. After excluding three survivors with missing death data, 349 with missing dietary data, and 478 with missing covariates data, 1367 stroke survivors finally remained for the analysis.

### Dietary assessment

Trained interviewers performed a 24-h dietary recall interview with participants via the Computer-Assisted Dietary Interview (CADI) system. The dietary recall interview seeks to determine the varieties and quantities of foods as well as beverages (encompassing all forms of water) consumed within the 24 h before the interview. Depending on the nutrient values of individual foods/beverages reported in the Food and Nutrient Database for Dietary Studies (FNDDS), the daily total nutrient intakes were calculated and provided in NHANES^25^. Because nutrient intakes are strongly correlated with energy intake, dietary cholesterol intake was adjusted for energy intake according to the nutrient density approach. The adjusted intake formula is: dietary cholesterol intake/energy intake * 1000^26^.

Consistent with previous research^27,28^, egg consumption was defined as the intake of whole eggs or foods predominantly consisting of whole eggs, with the exception of egg whites or yolks and non-chicken eggs. First, we identified eligible FNDDS food codes associated with whole eggs in “31 (Eggs)” and “32 (Egg mixtures)” groups. Second, we looked up the food ingredient information and obtained the weight portion of egg component in each food from FNDDS databases. Third, we obtained the daily egg-related food intakes of participants through dietary recall interviews. Finally, one’s egg consumption was detected by multiplying the daily intake by the egg proportion for each food, followed by summing the calculated egg intake of each food. For instance, if an individual’s daily consumption of foods 31,105,030, “Egg, whole, fried with oil (egg proportion: 93.2%)” and 32,103,030, “Egg salad, made with creamy dressing (egg proportion: 78.6%)” were 50 g and 100 g, respectively. The estimated egg intake was 125.2 g (50 g * 93.2% + 100 g * 78.6%). Given that the average weight of an egg is 50 g, we divided egg consumption into two groups: ≤ 1 egg/day and > 1 egg/day.

### Outcome ascertainment

The vital status of the NHANES participants was ascertained through linkage to the National Death Index (NDI) death certificate records using both deterministic alongside probabilistic approaches^29^. The death cause was categorized dependent on the International Classification of Diseases, 10th Revision (ICD-10). The primary outcome was all-cause mortality and the secondary outcome was cardiovascular mortality, which encompassed death resulting from heart and cerebrovascular diseases (ICD-10 codes I00-I09, I11, I13, I20-I51, and I60-I69). The follow-up time was evaluated as the period from the date of the dietary recall interview to the date of death or follow-up end (December 31, 2019).

### Covariate assessment

To reduce the impact of confounding bias, we selected necessary covariates provided by NHANES for analysis. The covariates consisted of three categories: sociodemographic, health-related, and dietary variables. Sociodemographic variables included age, sex, race, education level, marital status, and the family income-to-poverty ratio (PIR). Health-related variables contained body mass index (BMI), serum creatinine, smoking, drinking, hypertension, diabetes mellitus, hyperlipidemia, coronary heart disease, cancer, and statin therapy. Dietary variables were composed of energy, protein, saturated fatty acid (SFA), monounsaturated fatty acid (MUFA), polyunsaturated fatty acid (PUFA), and sodium intakes. Dietary intakes of protein, SFA, MUFA, and PUFA were expressed as percentages of total energy intake. Smoking was defined as smoking > 100 cigarettes ever. Drinking was characterized as consuming > 12 drinks in any one year. Hypertension was recognized as self-reported high blood pressure and systolic or diastolic blood pressure ≥ 140 or ≥ 90 mmHg, respectively^30^. Diabetes mellitus was recognized as self-reported high blood glucose, fasting blood glucose ≥ 126 mg/dL, or hemoglobin A1c ≥ 6.5%^31^. Hyperlipidemia was recognized as self-reported high cholesterol and low-density or non-high-density lipoprotein cholesterol ≥ 130 or ≥ 160 mg/dL, respectively^32^. The coronary heart disease and cancer diagnoses and statin therapy were determined based on self-reports of participants.

### Statistical analysis

The presentation form of continuous variables was the weighted median as well as the interquartile range (IQR), while the presentation form of categorial variables was the unweighted frequency alongside weighted percentage. The Spearman’s correlation coefficient was performed to explore the correlation between dietary cholesterol intake, egg consumption, and serum cholesterol concentration. Given the lack of established safety thresholds for dietary cholesterol intake in stroke populations, and to enhance the interpretability of our findings, we categorized dietary cholesterol intake into quartiles (Q1-Q4) for subsequent analyses.

Cox proportional hazards regression models were used to examine the relations of dietary cholesterol intake or egg consumption with risk of all-cause and cardiovascular mortality. Models were sequentially adjusted for age, sex, race, education level, marital status, and PIR (Model 1), plus energy, protein, SFA, MUFA, PUFA, and sodium intakes (Model 2), plus BMI, serum creatinine, smoking, drinking, hypertension, diabetes mellitus, hyperlipidemia, coronary heart disease, cancer, and statin therapy (Model 3). Although comorbidities such as hyperlipidemia may mediate the causal pathway between dietary intakes of cholesterol and egg and mortality, these conditions might also influence dietary choices, potentially introducing confounding bias. To address this, Model 2 initially adjusted for dietary variables, while the fully adjusted Model 3 additionally incorporated comorbidities including hyperlipidemia. Kaplan–Meier curves were constructed to visualize the survival status of the participants across intake groups of dietary cholesterol or eggs. To explore the potential nonlinear relationships of dietary cholesterol intake or egg consumption with risk of all-cause and cardiovascular mortality, we applied restricted cubic splines (RCS) regression models with four knots (5th, 35th, 65th, and 95th percentiles), setting the 50th percentile as the reference point^33^.

To evaluate effect modification by important demographic or clinical characteristics, exploratory subgroup analyses were performed considering the following variables: age (< 65, ≥ 65 years), sex (females, males), BMI (< 30, ≥ 30 kg/m^2^), hypertension (y/n), diabetes mellitus (y/n), hyperlipidemia (y/n), statin therapy (y/n). Interactions were detected on the multiplicative scale by introducing the product term of both variables into the model. Kaplan–Meier curves, restricted cubic splines regression models, and subgroup analyses were adjusted for the same covariates as in Model 3. All statistical analyses were performed using R software (version 4.2.1). Key R packages included: *survey* (v4.4-2), *gtsummary* (v2.0.4), *survival* (v3.8-3), *survminer* (v0.5.0), *rms* (v7.0-0), *epitools* (v0.5-10.1), and *Hmisc* (v5.2-2). Statistical significance was defined as a two-sided *p* < 0.05. All analyses accounted for NHANES’s complex multistage sampling design by applying sampling weights (1/5 * WTDR4YR for 1999–2002 or 1/10 * WTDRD1 for 2003–2018) to ensure national representativeness and incorporating clustering variables (SDMVPSU for primary sampling units) and stratification variables (SDMVSTRA)^34^.

## Results

### Participants characteristics

This study included 1367 stroke survivors with 9869.5 person-years of follow-up data. Median age was 66.0 years (IQR, 54.0–76.0) at baseline, 740 (72.7%) were non-Hispanic White, and 681 (57.7%) were females. Participants’ characteristics, according to the survival status, varied significantly in age, race, marital status, BMI, serum creatinine, hypertension, coronary heart disease, cancer, and dietary energy and cholesterol intakes (Table 1 and S1). Overall median dietary cholesterol intake was 119.0 mg/1000 kcal per day (IQR, 78.2–187.2) and 97 (16.5%) participants consumed > 1 egg per day. Tables S2–3 present participants’ characteristics between different dietary cholesterol intake or egg consumption groups. The incidence per 1000 person-years was 59.9 (95% confidence interval [CI], 55.2–64.9) for all-cause and 23.3 (95% CI, 20.4–26.5) for cardiovascular mortality. Figure S1 illustrates Spearman’s correlation of dietary cholesterol intake and egg consumption (*ρ* = 0.67, *P* < 0.001). Dietary cholesterol intake and egg consumption were generally not related to serum cholesterol concentration, and only relatively weak links between dietary cholesterol and serum total cholesterol (*ρ* = 0.094, *P* = 0.012) and non-high-density lipoprotein cholesterol (*ρ* = 0.079, *P* = 0.033) were noticed in participants without statin therapy (Table S4).

**Table 1:** Baseline characteristics of cohort participants according to survival status defined by all-cause mortality*.

	Overall (*n* = 1367)	Alive (*n* = 776)	Death (*n* = 591)
Age, y	66.0 (54.0–76.0)	61.0 (50.0–70.0)	75.0 (66.0–80.0)
Sex, %			
Female	681 (57.7)	430 (59.6)	251 (54.5)
Male	686 (42.3)	346 (40.4)	340 (45.5)
Race, %			
Mexican American	143 (4.1)	84 (4.8)	59 (2.9)
Other Hispanic	67 (2.8)	51 (3.4)	16 (1.9)
Non-Hispanic White	740 (72.7)	348 (67.5)	392 (81.2)
Non-Hispanic Black	340 (13.9)	235 (16.3)	105 (10.0)
Other Race	77 (6.5)	58 (8.0)	19 (4.0)
Education level, %			
High school or less	846 (58.4)	452 (53.3)	394 (66.7)
Some college	350 (24.5)	221 (27.4)	129 (19.8)
College graduate	171 (17.1)	103 (19.4)	68 (13.5)
Marital status, %			
Married/living with partner	733 (57.2)	430 (61.1)	303 (50.9)
Widowed/divorced/separated	538 (35.6)	268 (28.9)	270 (46.5)
Never married	96 (7.2)	78 (10.0)	18 (2.6)
PIR	1.9 (1.1–3.4)	2.1 (1.1–3.8)	1.8 (1.2–2.9)
BMI, kg/m2	29.0 (25.1–33.7)	29.9 (25.6–34.7)	27.9 (24.8–31.9)
Serum creatinine, µmol/L	84.0 (69.8–105.2)	79.6 (67.2–94.6)	97.2 (76.0–114.9)
Smoking, %	839 (59.9)	466 (59.2)	373 (61.1)
Drinking, %	829 (62.1)	461 (64.3)	368 (58.5)
Hypertension, %	1078 (75.6)	594 (72.5)	484 (80.7)
Diabetes mellitus, %	499 (32.9)	273 (31.3)	226 (35.7)
Hyperlipidemia, %	940 (68.9)	556 (70.2)	384 (66.7)
Coronary heart disease, %	247 (19.0)	114 (14.8)	133 (25.9)
Cancer, %	295 (21.3)	129 (16.3)	166 (29.6)
Statin therapy, %	650 (46.9)	385 (48.0)	265 (45.1)
Daily dietary intake			
Energy, kcal	1686.7 (1238.9–2236.1)	1793.2 (1296.7–2323.0)	1548.6 (1137.2–2013.5)
Protein, % energy	14.9 (12.2–18.1)	14.7 (12.0–18.1)	15.1 (12.4–18.2)
Fat, % energy	34.7 (29.1–40.8)	35.1 (28.9–41.9)	34.1 (29.3–39.4)
SFA, % energy	11.2 (8.8–13.8)	11.6 (8.8–14.3)	11.0 (8.9–13.6)
MUFA, % energy	12.2 (9.9–15.0)	12.3 (9.7–15.0)	12.2 (10.2–14.7)
PUFA, % energy	7.2 (5.3–9.3)	7.2 (5.3–9.3)	7.2 (5.4–9.3)
Sodium, mg/1000 kcal	1614.6 (1312.5–1985.8)	1638.1 (1326.3–1989.2)	1594.5 (1297.5–1980.3)
Cholesterol, mg/1000 kcal	119.0 (78.2–187.2)	115.9 (75.3–175.5)	124.4 (83.4–214.6)
> 1 egg, %	224 (15.0%)	127 (14.1%)	97 (16.5%)

BMI, body mass index; MUFA, monounsaturated fatty acid; PIR, family income-to-poverty ratio. PUFA, polyunsaturated fatty acid; SFA, saturated fatty acid.

* The descriptive analyses in Table 1 accounted for the complex multistage sampling weights.

### Dietary cholesterol intake and mortality

**Fig. 2:**
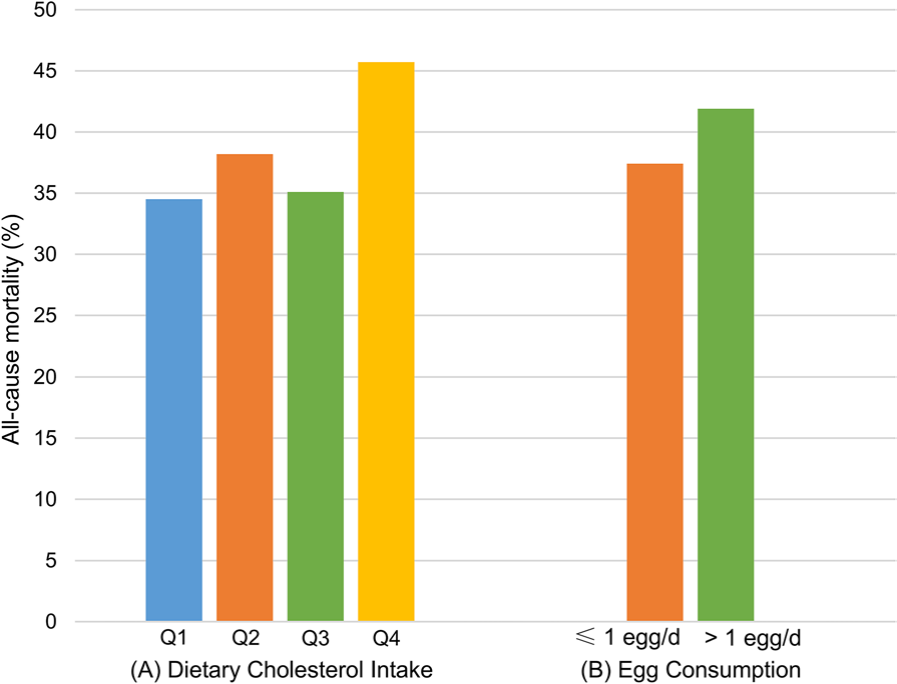
The prevalence of all-cause mortality across different intake groups of (**A**) dietary cholesterol and (**B**) eggs.

**Fig. 3:**
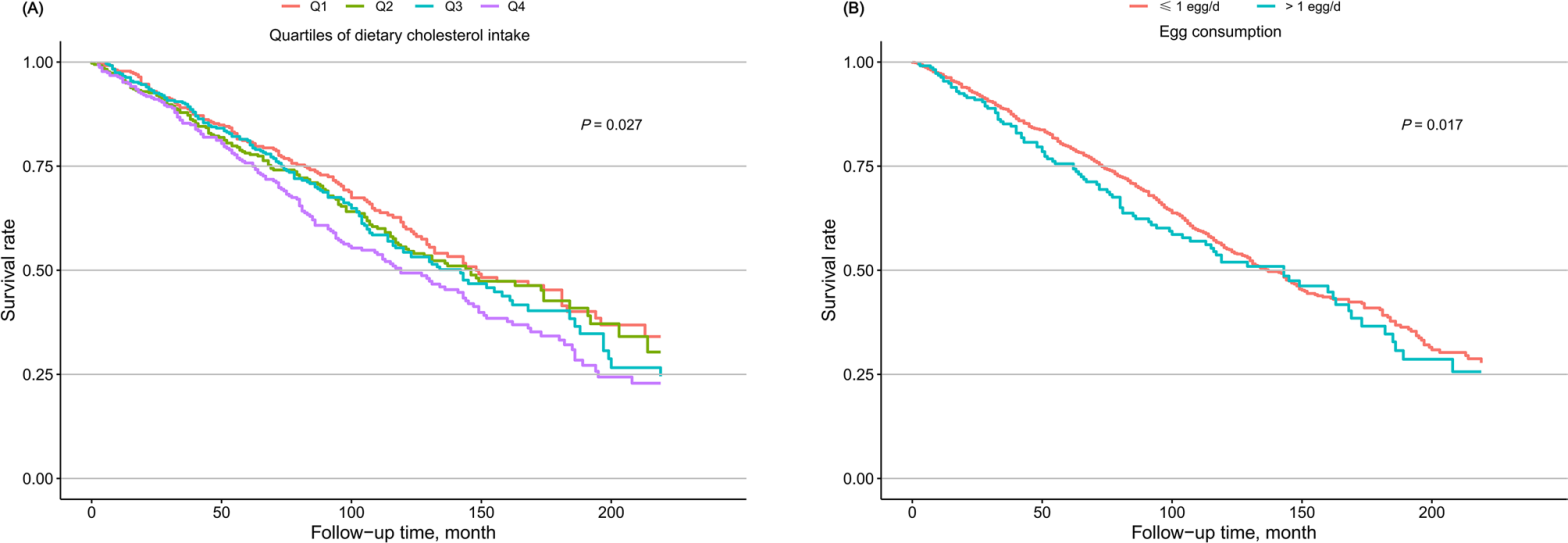
The Kaplan–Meier curve of all-cause mortality for the study participants with different (**A**) dietary cholesterol intake and (**B**) egg consumption.

**Fig. 4:**
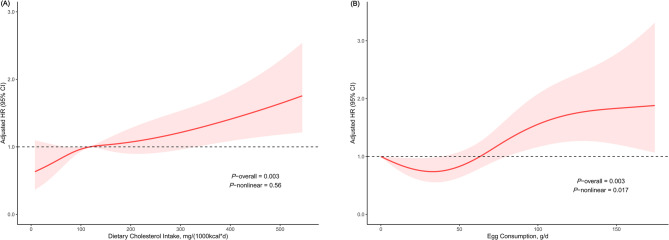
Restricted cubic spline regression model of the associations of (**A**) dietary cholesterol intake and (**B**) egg consumption with risk of all-cause mortality after stroke.

After a median follow-up of 79.0 months (IQR, 40.0-131), ultimately, there were 591 all-cause deaths and 230 cardiovascular deaths. The prevalence of all-cause and cardiovascular mortality across different intake groups of dietary cholesterol was depicted in Figs. 2 and S2. For both all-cause and cardiovascular death outcomes, the survival rate of participants was significantly lowered in the Q4 group of dietary cholesterol intake compared with the other three groups (Figs. 3 and S3). Based on Model 3, each 100 mg/1000 kcal increase in dietary cholesterol consumption per day significantly increased the risk of all-cause (hazard ratio [HR], 1.16; 95% CI, 1.05–1.27; Table 2) and cardiovascular mortality (HR, 1.15; 95% CI, 1.00–1.31; Table 3). Participants in the Q4 group of dietary cholesterol intake had 66% and 80% higher risk of all-cause (HR, 1.66; 95% CI, 1.16–2.38; Table 2) and cardiovascular mortality (HR, 1.80; 95% CI, 0.98–3.60; Table 3), respectively, compared with those in the Q1 group. The RCS model elucidated that the increase in all-cause and cardiovascular mortality varied with dietary cholesterol intake in a significant linear manner (*P* for nonlinearity > 0.05; Figs. 4 and S4). In Model 3, dietary intakes of SFA, MUFA, and PUFA showed no significant associations with all-cause or cardiovascular disease mortality.

**Table 2:** Associations of dietary cholesterol intake and egg consumption with all-cause mortality.

	Death/Pearson-years	Unadjusted Model		Model 1		Model 2		Model 3	
		HR (95% CI)	*P*-value	HR (95% CI)	*P*-value	HR (95% CI)	*P*-value	HR (95% CI)	*P*-value
Dietary cholesterol intake									
Per 100-mg/1000 kcal/day increase		1.13 (1.05–1.22)	< 0.001	1.09 (1.02–1.18)	0.017	1.15 (1.05–1.25)	0.002	1.16 (1.05–1.27)	0.003
Quartiles									
Q1: ≤ 78.3 mg/1000 kcal/day	137/2442.0	Ref		Ref		Ref		Ref	
Q2: 78.4–118.1 mg/1000 kcal/day	145/2567.9	1.01 (0.75–1.37)	0.94	0.96 (0.72–1.29)	0.80	1.06 (0.78–1.44)	0.72	1.15 (0.85–1.56)	0.35
Q3: 118.2–203.8 mg/1000 kcal/day	151/2451.3	1.01 (0.76–1.32)	0.96	0.99 (0.69–1.41)	0.94	1.11 (0.74–1.68)	0.61	1.07 (0.70–1.63)	0.76
Q4: ≥ 203.9 mg/1000 kcal/day	158/2408.3	1.36 (1.05–1.76)	0.021	1.29 (0.99–1.69)	0.059	1.53 (1.08–2.17)	0.016	1.66 (1.16–2.38)	0.006
*P* for trend		0.025		0.084		0.028		0.027	
Egg consumption									
≤ 1 egg/day	494/8334.9	Ref		Ref		Ref		Ref	
> 1 egg/day	97/1534.6	1.12 (0.86–1.47)	0.40	1.30 (1.02–1.65)	0.032	1.38 (1.08–1.76)	0.010	1.40 (1.06–1.84)	0.017

Model 1was adjusted for age, sex, race, education level, marital status, and PIR;.

Model 2 was further adjusted for energy, protein, SFA, MUFA, PUFA, and sodium intakes;.

Model 3 was further adjusted for BMI, serum creatinine, smoking, drinking, hypertension, diabetes mellitus, hyperlipidemia, coronary heart disease, cancer, and statin therapy.

CI, confidence interval; HR, hazard ratio.

**Table 3:** Associations of dietary cholesterol intake and egg consumption with cardiovascular mortality.

	Death/Pearson-years	Unadjusted Model		Model 1		Model 2		Model 3	
		HR (95% CI)	*P*-value	HR (95% CI)	*P*-value	HR (95% CI)	*P*-value	HR (95% CI)	*P*-value
Dietary cholesterol intake									
Per 100-mg/1000 kcal/day increase		1.13 (1.01–1.26)	0.027	1.09 (0.98–1.21)	0.12	1.14 (1.01–1.30)	0.042	1.15 (1.00–1.31)	0.043
Quartiles									
Q1: ≤ 78.0 mg/1000 kcal/day	51/2391.0	Ref		Ref		Ref		Ref	
Q2: 78.1–116.5 mg/1000 kcal/day	57/2531.6	1.15 (0.74–1.79)	0.53	1.15 (0.74–1.81)	0.53	1.30 (0.77–2.22)	0.33	1.33 (0.76–2.34)	0.32
Q3: 116.6–200.9 mg/1000 kcal/day	60/2475.8	1.12 (0.71–1.77)	0.62	1.12 (0.63–1.98)	0.70	1.30 (0.66–2.56)	0.44	1.20 (0.62–2.31)	0.59
Q4: ≥ 201.0 mg/1000 kcal/day	62/2471.1	1.39 (0.90–2.15)	0.14	1.43 (0.96–2.13)	0.082	1.78 (0.98–3.24)	0.058	1.80 (0.98–3.60)	0.059
*P* for trend		0.18		0.14		0.079		0.082	
Egg consumption									
≤ 1 egg/day	189/8334.9	Ref		Ref		Ref		Ref	
> 1 egg/day	41/1534.6	1.13 (0.75–1.69)	0.56	1.33 (0.86–2.05)	0.20	1.35 (0.88–2.08)	0.17	1.47 (0.93–2.31)	0.10

Model 1was adjusted for age, sex, race, education level, marital status, and PIR;.

Model 2 was further adjusted for energy, protein, SFA, MUFA, PUFA, and sodium intakes;.

Model 3 was further adjusted for BMI, serum creatinine, smoking, drinking, hypertension, diabetes mellitus, hyperlipidemia, coronary heart disease, cancer, and statin therapy.

CI, confidence interval; HR, hazard ratio.

### Egg consumption and mortality

The prevalence of all-cause and cardiovascular mortality across different intake groups of eggs was depicted in Figs. 2 and S2. For all-cause death outcomes, the participant survival rate was significantly lowered in the group consuming > 1 egg/day compared with the group consuming ≤ 1 egg/day (Fig. 3). Nonetheless, no significant variance was recognized in the survival rate for cardiovascular death outcomes between these two groups (Figure S3). Based on model 3, participants consuming > 1 egg/day had a 40% increased risk of all-cause mortality (HR, 1.40; 95% CI, 1.06–1.84; Table 2) compared with those consuming ≤ 1 egg/day. However, the association between egg consumption and cardiovascular mortality (HR, 1.47; 95% CI, 0.93–2.31; Table 3) was not statistically significant. The RCS model showed a significant nonlinear relationship between egg consumption and all-cause mortality (*P* for nonlinearity = 0.018; Fig. 4). Taking no egg consumption per day as a reference, the risk of all-cause mortality was decreased until around 33.3 g/day of egg consumption and then started to increase afterward, reaching a significant difference at 82.3 g/day. The RCS model did not find a possible nonlinear relationship between egg consumption and cardiovascular mortality (Figure S4).

### Subgroup analyses

**Fig. 5:**
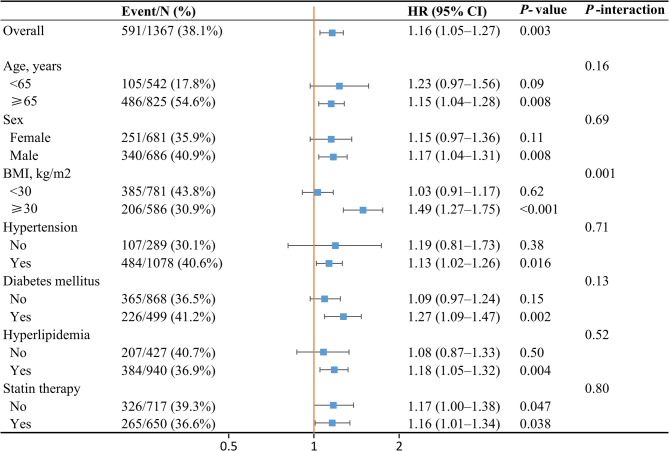
Association between dietary cholesterol intake and risk of all-cause mortality among different subgroups.

The association between dietary cholesterol intake (per 100 mg/1000 kcal/day) and all-cause mortality was stronger in participants aged ≥ 65 years (HR, 1.15; 95% CI, 1.04–1.28), males (HR, 1.17; 95% CI, 1.04–1.31), and those with BMI ≥ 30 (HR, 1.49; 95% CI, 1.27–1.75), hypertension (HR, 1.13; 95% CI, 1.02–1.26), diabetes mellitus (HR, 1.27; 95% CI, 1.09–1.47), and hyperlipidemia (HR, 1.18; 95% CI, 1.05–1.32) (Fig. 5). A significant interaction between dietary cholesterol intake and BMI (*P* for interaction = 0.001) was observed. The association between dietary cholesterol intake and cardiovascular mortality was stronger in participants with BMI ≥ 30 (HR, 1.41; 95% CI, 1.11–1.80) and those without hypertension (HR, 1.75; 95% CI, 1.27–2.41) (Figure S5). A nearly statistically significant interaction between dietary cholesterol intake and BMI (*P* for interaction = 0.072) was observed.

**Fig. 6:**
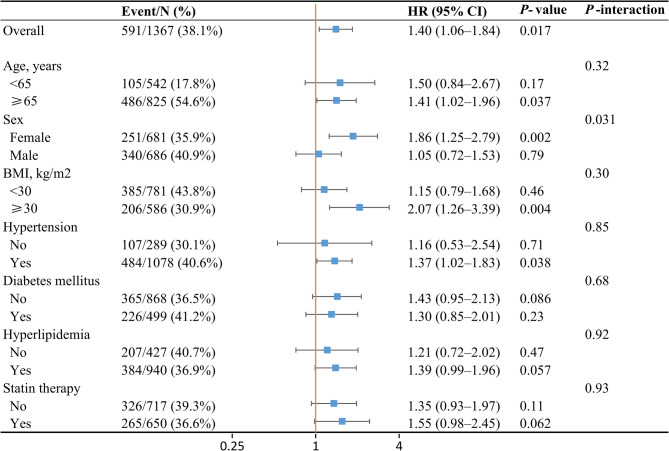
Association between egg consumption and risk of all-cause mortality among different subgroups.

The association between consuming more than one egg per day and all-cause mortality was stronger in participants aged ≥ 65 years (HR, 1.41; 95% CI, 1.02–1.96), females (HR, 1.86; 95% CI, 1.25–2.79), and those with BMI ≥ 30 (HR, 2.07; 95% CI, 1.26–3.39), and hypertension (HR, 1.37; 95% CI, 1.02–1.83) (Fig. 6). A significant interaction between egg consumption and sex (*P* for interaction = 0.031) was observed. The association between consuming more than one egg per day and cardiovascular mortality was stronger in participants without hypertension (HR, 6.55; 95% CI, 3.23–13.3) and those with hyperlipidemia (HR, 1.66; 95% CI, 1.04–2.64) (Figure S6). A significant interaction between egg consumption and sex was also recognized (*P* for interaction = 0.007).

## Discussion

Among 1367 stroke survivors from this prospective survey in the US with a median follow-up of 6.6 years, higher dietary cholesterol intake was significantly associated with escalated risk of all-cause and cardiovascular mortality, in a dose-response manner. Egg consumption possessed a nonlinear relationship with risk of all-cause mortality. Although the positive trend between egg consumption and cardiovascular mortality did not reach statistical significance, caution is warranted regarding potential Type II error due to limited outcome event or competing risks of other causes of death.

The all-cause mortality rate per 1000 person-years of stroke survivors in this study was apparently higher than that of healthy older people in a previous multicenter cohort (59.9 vs. 11.9)^35^. Thus, it is worthwhile to clarify the lifestyles that affect the mortality rate of stroke survivors, such as daily consumption of dietary cholesterol and eggs. A dose-response meta-analysis has evaluated 14 cohort studies that examined the associations of dietary cholesterol with all-cause and cardiovascular mortality among the community population^18^. Outcomes of the meta-analysis reported a significant nonlinear association between dietary cholesterol intake with risk of all-cause mortality (pooled relative risk [RR], 1.06; 95% CI, 1.03–1.08) and no association with risk of cardiovascular mortality. More recent results from large prospective cohorts suggested positive associations between dietary cholesterol intake and risk of all-cause and cardiovascular mortality^13,36^, similar to findings of the current study. However, Pan et al. reported a nonlinear positive relationship between dietary cholesterol intake and risk of all-cause mortality in Black Americans, a null link in White Americans, and a U-shaped association in Chinese^22^. By distinguishing the dietary sources of cholesterol, Zhuang et al. found that egg-sourced cholesterol was correlated with a reduced risk of all-cause mortality, while non-egg-sourced cholesterol was associated with raised risk of all-cause mortality^14^. Egg consumption has gained more intense attention as a major source of dietary cholesterol. In a recent published meta-analysis that combined 25 cohort studies, egg consumption was not associated with risk of all-cause and cardiovascular mortality among the community population^19^. However, subsequent subgroup analyses reported positive associations between egg consumption and risk of all-cause and cardiovascular mortality across studies performed in the US; further dose-response meta-analysis revealed that an additional intake of 1 egg/week was related to a 2% increased risk of all-cause mortality in a linear manner (pooled RR, 1.02; 95% CI, 1.00–1.03), but not with risk of cardiovascular mortality^19^. Divided by the region where the studies were conducted, the association between egg consumption and risk of all-cause mortality with the largest sample was U-shaped in East Asia (*n* = 134,280)^22^, negatively correlated in West Asia (*n* = 42,403)^37^, negatively correlated in Europe (*n* = 120,852)^38^, and positively correlated in North America (*n* = 521,120)^12^. The inconsistent outcomes across the aforementioned studies may be attributed to diverse population features (e.g., sociodemographic characteristics, dietary structure, lifestyles), methods of dietary assessment and death ascertainment, adjustments for confounders, and duration of follow-up.

Herein, we proposed several potential mechanisms to explain the associations of consumption of dietary cholesterol and eggs with all-cause and cardiovascular mortality. First, increased cholesterol intake is accompanied by an increase in serum total cholesterol level^13^, which leads to the formation and progression of atherosclerotic plaque and ultimately promotes risk of death from cardiovascular diseases^13,39^. Nonetheless, consistent with our findings, the correlation between dietary and serum cholesterol is extremely weak due to the regulatory mechanism of cholesterol absorption by intestinal cells and synthesis by liver cells^40^. It is noted that dietary cholesterol intake has more pronounced influences on elevating blood total cholesterol, low-density lipoprotein cholesterol, and non-high-density lipoprotein cholesterol levels in persons with high genetic susceptibility for hypercholesteremia^41^. For example, the ApoE polymorphism influences cholesterol absorption efficiency, synthesis rates, and LDL apoB clearance, thereby modulating individual responsiveness to dietary cholesterol^42^. Second, a high-cholesterol diet leads to alterations in the gut microbiota and microbial metabolites, and in turn, unabsorbed cholesterol is metabolized by gut microbiota into coprostanol and cholesterol-3-sulfate^43^. The interaction between dietary cholesterol and gut microbiota exerts influences on host health, including colorectal cancer and ulcerative colitis. However, it is not clear whether gut microbiota mediates the adverse impacts of high cholesterol intake on mortality. Third, individuals with high consumption of dietary cholesterol or eggs tend to have unhealthy lifestyles, including physical inactivity, current smoking, and unhealthy dietary patterns (e.g., higher red meat and lower fruit and vegetable intakes) in the US^11,12^. These unhealthy lifestyles are important components of the Life’s Essential 8 and closely relate to all-cause and cardiovascular mortality in adults^44^. By comparison, high consumption of dietary cholesterol or egg was associated with increased amounts of physical activity and intakes of fruits and vegetables in Europe^13,45^ and China^14,20^. This diversity of accompanying lifestyles partly accounts for the discrepancy in the associations of consumption of dietary cholesterol and eggs with all-cause and cardiovascular mortality across different regions. Fourth, unlike the linear increase in risk of all-cause mortality due to dietary cholesterol intake, egg consumption showed a significant increase in risk of all-cause mortality after exceeding 80.6 g, while consumption below this level was safe and even beneficial. The double-edged sword effect of eggs depends on their components of multiple nutrients, not only fat and cholesterol, but also abundant protein, minerals, and vitamins^6^. Extensive egg-sourced fat intake has been reported to raise the absolute risk of all-cause and cardiovascular mortality by 1.40% and 0.82%, respectively^46^. Dietary cholesterol explains 43.0–63.2% and 39.3–62.3% of the association of egg consumption with all-cause^12^ and cardiovascular mortality^47^, respectively. In a prospective cohort study of 521,120 US adults with median 16-year follow-up, consumption of egg whites/substitutes showed protective effects against all-cause mortality after removing cholesterol-rich egg yolks^12^. Since egg whites are low in fat and cholesterol yet preserve most other essential egg nutrients, they avoid the double-edged sword effect associated with whole egg consumption.

Subgroup analyses provide more comprehensive insights into the associations of consumption of dietary cholesterol and eggs with all-cause and cardiovascular mortality. The associations tend to be intensified in persons with cardiometabolic diseases (obesity, hypertension, diabetes mellitus, hyperlipidemia) or risk factors of cardiometabolic diseases (advanced age). In populations with cardiometabolic diseases, more frequent unhealthy lifestyles^48^ and metabolic disorder, including insulin resistance and dyslipidemia^49^, are not conducive to maintaining cholesterol homeostasis and may cause the amplification of the adverse impacts of consumption of dietary cholesterol and eggs. The stronger association of dietary cholesterol intake and egg consumption with cardiovascular mortality in the non-hypertension subgroup needs to be interpreted with caution. The Type I error derived from selection bias may exist in view of the small sample size of the non-hypertension subgroup. It is unclear why there are different manifestations of dietary cholesterol intake and egg consumption in subgroups stratified by sex. However, this reveals that the effects of dietary cholesterol and eggs on human health are not entirely consistent.

This study, as far as we know, is the first cohort study on the associations of dietary cholesterol intake and egg consumption with all-cause and cardiovascular mortality in the population with stroke. Furthermore, the RCS models and subgroup analyses are important strengths of our study depicting the nonlinear relationship between egg consumption and all-cause mortality and screening high-risk subgroups for dietary cholesterol intake and egg consumption. However, statistical power was limited in some subgroups due to insufficient sample sizes or low event frequencies. Our study also has several limitations. First, the research sample for this study is restricted to individuals from the US. Due to the inevitable differences in population characteristics, the findings of this study cannot be extrapolated to populations in other regions. Second, the determination of stroke diagnosis is based on participants’ self-reports rather than medical records. This has a potential risk of recall bias and fails to discriminate between ischemic and hemorrhagic stroke, which have differential risk factor profiles and short-term mortality risk^50^. Third, this study does not distinguish the food sources of dietary cholesterol, which has shown different effects on mortality in the Chinese population^14^. Fourth, 24-h dietary recalls cannot reflect the long-term exposure levels of egg consumption. Thus, using a dichotomous variable (> 1/≤ 1 egg per day) rather than a continuous variable, we evaluated the effect of egg consumption on mortality to reduce information bias. Fifth, we did not distinguish the cooking methods of eggs, which may change the nutrient content of eggs. Nonetheless, there is no evidence that changes in cooking methods affect the association between egg consumption and mortality^12^. Sixth, due to the limited variables available in NHANES, residual confounding has not been fully eliminated, as variables such as fruit and vegetable intake and physical activity were not accounted for. Further randomized controlled trials are required to elucidate the long-term influences of consumption of dietary cholesterol and eggs on mortality.

## Conclusions

In a modest sample of US stroke survivors, we found significant positive associations between dietary cholesterol intake and risk of all-cause and cardiovascular mortality, whereas egg consumption was related to risk of all-cause mortality in a nonlinear manner. Our findings indicate that limiting dietary cholesterol intake and moderate consumption of up to 1 egg per day might be beneficial for the health of stroke survivors. However, given the limitations of this study, our analyses should be replicated in larger cohorts, more rigorous designs, and populations in other regions before the formulation of clinical practice and public health guidelines to enhance the long-term health and longevity of patients with stroke.

## Supplementary Information

Below is the link to the electronic supplementary material.

Supplementary Material 1

## Data Availability

The datasets generated and analysed during the current study are available from the corresponding author on reasonable request.
