# Supplementary material for: Dietary cholesterol intake and egg consumption in relation to all-cause and cardiovascular mortality after stroke
Source: Sci Rep. 2025 Oct 8;15:35163. doi: 10.1038/s41598-025-19028-0 (PMC12508433; doi:10.1038/s41598-025-19028-0)
Supplement: Supplementary file 1 — Supplementary Material 1 [file 41598_2025_19028_MOESM1_ESM.docx]

**Dietary Cholesterol Intake and Egg Consumption in Relation to All-Cause and Cardiovascular Mortality after Stroke**

**Table S1.** Baseline characteristics of cohort participants according to survival status defined by cardiovascular mortality

**Table S2.** Baseline characteristics of cohort participants according to the quartiles of dietary cholesterol intake

**Table S3.** Baseline characteristics of cohort participants according to egg consumption

**Table S4.** Spearman correlation coefficients of dietary cholesterol intake and egg consumption with serum cholesterol concentration

**Figure S1.** Spearman correlation coefficients between dietary cholesterol intake and egg consumption

**Figure S2.** The prevalence of cardiovascular mortality across different intake groups of (A) dietary cholesterol and (B) eggs.

**Figure S3.** The Kaplan–Meier curve of cardiovascular mortality for the study participants with different (A) dietary cholesterol intake and (B) egg consumption

**Figure S4.** Restricted cubic spline regression model of the associations of (A) dietary cholesterol intake and (B) egg consumption with risk of cardiovascular mortality after stroke.

**Figure S5.** Association between dietary cholesterol intake and risk of cardiovascular mortality among different subgroups

**Figure S6.** Association between egg consumption and risk of cardiovascular mortality among different subgroups

**Table S1.** Baseline characteristics of cohort participants according to survival status defined by cardiovascular mortality

|  | **Alive (n=776)** | **Death (n=230)** |
| --- | --- | --- |
| Age, y | 61.0 (50.0–70.0) | 76.0 (66.0–80.0) |
| Sex, % |  |  |
| Female | 430 (59.6) | 90 (53.5) |
| Male | 346 (40.4) | 140 (46.5) |
| Race, % |  |  |
| Mexican American | 84 (4.8) | 27 (3.5) |
| Other Hispanic | 51 (3.4) | 5 (0.8) |
| Non-Hispanic White | 348 (67.5) | 152 (82.4) |
| Non-Hispanic Black | 235 (16.3) | 41 (10.1) |
| Other Race | 58 (8.0) | 5 (3.2) |
| Education level, % |  |  |
| High school or less | 452 (53.3) | 139 (59.3) |
| Some college | 221 (27.4) | 56 (23.1) |
| College graduate | 103 (19.4) | 35 (17.7) |
| Marital status, % |  |  |
| Married/living with partner | 430 (61.1) | 123 (53.5) |
| Widowed/divorced/separated | 268 (28.9) | 102 (45.3) |
| Never married | 78 (10.0) | 5 (1.2) |
| PIR | 2.1 (1.1–3.8) | 1.8 (1.2–2.9) |
| BMI, kg/m2 | 29.9 (25.6–34.7) | 27.2 (24.6–31.5) |
| Serum creatinine, μmol/L | 79.6 (67.2–94.6) | 97.2 (74.3–114.9) |
| Smoking, % | 466 (59.2) | 137 (58.8) |
| Drinking, % | 461 (64.3) | 136 (55.1) |
| Hypertension, % | 182 (27.5) | 40 (20.1) |
| Diabetes mellitus, % | 273 (31.3) | 79 (30.3) |
| Hyperlipidemia, % | 556 (70.2) | 153 (69.7) |
| Coronary heart disease, % | 114 (14.8) | 58 (30.7) |
| Cancer, % | 129 (16.3) | 62 (31.3) |
| Statin therapy, % | 385 (48.0) | 107 (45.8) |
| Daily dietary intake |  |  |
| Energy, kcal | 1793.2 (1296.7–2323.0) | 1530.9 (1234.3–1984.9) |
| Protein, % energy | 14.9 (12.1–18.1) | 14.7 (12.0–18.1) |
| Fat, % energy | 35.1 (28.9–41.9) | 34.9 (29.7–39.0) |
| SFA, % energy | 11.3 (8.7–13.9) | 11.6 (8.8–14.3) |
| MUFA, % energy | 12.3 (9.9–15.1) | 12.3 (9.7–15.0) |
| PUFA, % energy | 7.2 (5.3–9.4) | 7.2 (5.3–9.3) |
| Sodium, mg/1000 kcal | 1638.1 (1326.3–1989.2) | 1545.0 (1266.6–2040.3) |
| Cholesterol, mg/1000 kcal | 115.9 (75.3–175.0) | 118.2 (81.6–190.7) |
| > 1 egg, % | 127 (14.1) | 41 (16.6) |

BMI, body mass index; MUFA, monounsaturated fatty acid; PIR, family income-to-poverty ratio. PUFA, polyunsaturated fatty acid; SFA, saturated fatty acid.

**Table S2.** Baseline characteristics of cohort participants according to the quartiles of dietary cholesterol intake

|  | **Q1 (n=342 )** | **Q2 (n=341 )** | **Q3 (n=342 )** | **Q4 (n=342 )** |
| --- | --- | --- | --- | --- |
| Age, y | 65.0 (52.9–76.0) | 68.0 (53.8–77.0) | 65.0 (55.0–74.0) | 67.0 (56.0–77.0) |
| Sex, % |  |  |  |  |
| Female | 182 (60.5) | 174 (59.0) | 167 (55.3) | 158 (56.2) |
| Male | 160 (39.5) | 167 (41.0) | 175 (44.7) | 184 (43.8) |
| Race, % |  |  |  |  |
| Mexican American | 35 (3.7) | 30 (3.1) | 29 (3.3) | 49 (6.6) |
| Other Hispanic | 23 (3.3) | 14 (2.1) | 15 (3.0) | 15 (2.7) |
| Non-Hispanic White | 186 (75.1) | 195 (73.1) | 190 (72.9) | 169 (69.4) |
| Non-Hispanic Black | 76 (11.3) | 76 (13.5) | 94 (14.1) | 94 (16.9) |
| Other Race | 22 (6.6) | 26 (8.2) | 14 (6.6) | 15 (4.2) |
| Education level, % |  |  |  |  |
| High school or less | 210 (54.1) | 205 (59.3) | 218 (60.9) | 213 (59.0) |
| Some college | 83 (24.7) | 94 (23.8) | 86 (23.6) | 87 (26.1) |
| College graduate | 49 (21.2) | 42 (16.8) | 38 (15.5) | 42 (14.9) |
| Marital status, % |  |  |  |  |
| Married/living with partner | 171 (54.9) | 182 (55.0) | 189 (59.3) | 191 (59.6) |
| Widowed/divorced/separated | 146 (36.3) | 138 (39.5) | 126 (32.8) | 128 (34.2) |
| Never married | 25 (8.8) | 21 (5.5) | 27 (7.9) | 23 (6.2) |
| PIR | 1.8 (1.0–3.1) | 2.0 (1.2–3.8) | 2.1 (1.2–3.6) | 1.8 (1.1–3.1) |
| BMI, kg/m2 | 28.3 (24.4–33.2) | 29.1 (25.3–33.8) | 29.1 (25.2–34.2) | 29.6 (25.8–34.0) |
| Serum creatinine, μmol/L | 84.9 (69.0–102.2) | 84.0 (67.1–100.8) | 81.3 (70.3–106.1) | 88.4 (70.7–106.1) |
| Smoking, % | 219 (62.2) | 205 (56.8) | 222 (63.7) | 193 (56.1) |
| Drinking, % | 208 (63.8) | 200 (60.1) | 215 (61.3) | 206 (63.4) |
| Hypertension, % | 256 (72.2) | 262 (75.4) | 275 (74.0) | 285 (81.6) |
| Diabetes mellitus, % | 118 (31.0) | 105 (28.7) | 121 (30.9) | 155 (42.4) |
| Hyperlipidemia, % | 225 (68.0) | 237 (70.2) | 226 (64.5) | 252 (73.9) |
| Coronary heart disease, % | 60 (18.6) | 61 (19.5) | 59 (18.5) | 67 (19.6) |
| Cancer, % | 72 (21.1) | 79 (24.7) | 79 (22.6) | 65 (16.4) |
| Statin therapy, % | 152 (43.8) | 165 (50.2) | 158 (45.2) | 175 (49.0) |
| Daily dietary intake |  |  |  |  |
| Energy, kcal | 1559.0 (1125.0–2149.9) | 1756.6 (1304.9–2208.1) | 1815.9 (1377.6–2357.0) | 1575.2 (1143.7–2004.0) |
| Protein, % energy | 12.2 (9.5–14.3) | 14.3 (12.2–17.1) | 16.5 (13.5–19.8) | 17.0 (14.6–20.5) |
| Fat, % energy | 30.4 (23.5–36.3) | 34.0 (29.3–39.6) | 36.1 (31.0–41.0) | 37.5 (33.2–43.7) |
| SFA, % energy | 9.3 (6.8–12.0) | 11.9 (9.0–14.7) | 11.5 (9.5–14.5) | 12.0 (9.8–14.3) |
| MUFA, % energy | 10.3 (8.1–13.4) | 11.8 (9.8–14.5) | 12.7 (10.8–15.0) | 13.7 (11.6–16.4) |
| PUFA, % energy | 7.0 (5.1–9.4) | 6.6 (4.4–9.0) | 7.2 (5.4–9.4) | 7.8 (5.9–9.3) |
| Sodium, mg/1000 kcal | 1461.1 (1151.1–1813.0) | 1562.6 (1282.2–1934.0) | 1637.3 (1350.1–1980.3) | 1824.4 (1488.4–2156.9) |
| > 1 egg, % | 0 (0.0) | 3 (1.4) | 28 (7.6) | 203 (59.2) |
| All-cause Death, % | 137 (34.5) | 145 (38.2) | 151 (35.1) | 158 (45.7) |

BMI, body mass index; MUFA, monounsaturated fatty acid; PIR, family income-to-poverty ratio. PUFA, polyunsaturated fatty acid; SFA, saturated fatty acid.

**Table S3.** Baseline characteristics of cohort participants according to egg consumption

|  | **≤ 1 egg/d**  **(n=1143)** | **> 1 egg/d (n=224)** |
| --- | --- | --- |
| Age, y | 66.0 (54.0–76.0) | 63.0 (55.0–75.0) |
| Sex, % |  |  |
| Female | 599 (59.5) | 82 (47.3) |
| Male | 544 (40.5) | 142 (52.7) |
| Race, % |  |  |
| Mexican American | 107 (3.4) | 36 (8.2) |
| Other Hispanic | 57 (2.4) | 10 (5.0) |
| Non-Hispanic White | 633 (74.2) | 107 (64.3) |
| Non-Hispanic Black | 276 (13.2) | 64 (17.7) |
| Other Race | 70 (6.8) | 7 (4.7) |
| Education level, % |  |  |
| High school or less | 706 (57.8) | 140 (61.4) |
| Some college | 293 (24.5) | 57 (24.6) |
| College graduate | 144 (17.7) | 27 (14.0) |
| Marital status, % |  |  |
| Married/living with partner | 599 (55.5) | 134 (66.8) |
| Widowed/divorced/separated | 463 (37.3) | 75 (25.9) |
| Never married | 81 (7.1) | 15 (7.3) |
| PIR | 1.9 (1.1–3.4) | 1.9 (1.1–3.4) |
| BMI, kg/m2 | 28.9 (25.0–33.7) | 30.0 (25.5–34.5) |
| Serum creatinine, μmol/L | 84.0 (69.8–105.2) | 84.0 (70.7–105.6) |
| Smoking, % | 708 (60.4) | 131 (57.5) |
| Drinking, % | 676 (60.5) | 153 (71.1) |
| Hypertension, % | 895 (75.4) | 183 (76.7) |
| Diabetes mellitus, % | 395 (31.2) | 104 (42.6) |
| Hyperlipidemia, % | 779 (68.2) | 161 (72.5) |
| Coronary heart disease, % | 202 (19.0) | 45 (19.3) |
| Cancer, % | 256 (22.3) | 39 (16.0) |
| Statin therapy, % | 536 (46.1) | 114 (51.3) |
| Daily dietary intake |  |  |
| Energy, kcal | 1638.9 (1197.7–2197.4) | 1895.1 (1417.2–2467.1) |
| Protein, % energy | 14.7 (12.0–18.0) | 16.1 (13.3–19.4) |
| Fat, % energy | 34.1 (28.8–39.9) | 36.9 (32.5–43.8) |
| SFA, % energy | 11.0 (8.6–13.8) | 11.9 (9.9–13.9) |
| MUFA, % energy | 12.0 (9.7–14.7) | 13.5 (11.4–16.0) |
| PUFA, % energy | 7.2 (5.1–9.3) | 7.9 (5.9–9.8) |
| Sodium, mg/1000 kcal | 1599.2 (1289.0–1977.4) | 1686.4 (1421.9–2032.3) |
| Cholesterol, mg/1000 kcal | 109.3 (73.0–150.0) | 293.3 (237.8–367.4) |
| All-cause Death, % | 494 (37.4) | 97 (41.9) |

BMI, body mass index; MUFA, monounsaturated fatty acid; PIR, family income-to-poverty ratio. PUFA, polyunsaturated fatty acid; SFA, saturated fatty acid.

**Table S4.** Spearman correlation coefficients of dietary cholesterol intake and egg consumption with serum cholesterol concentration

|  | **Dietary cholesterol, mg/(1000kcal*d)** | | **Egg, g/d** | |
| --- | --- | --- | --- | --- |
|  | **Coefficient** | ***P* value** | **Coefficient** | ***P* value** |
| ***Statin therapy：Yes (n = 650)*** | | | | |
| Total cholesterol, mg/dL | 0.042 | 0.29 | 0.021 | 0.60 |
| High-density lipoprotein cholesterol, mg/dL | 0.042 | 0.29 | 0.042 | 0.28 |
| Non-high-density lipoprotein cholesterol, mg/dL | 0.024 | 0.54 | -0.004 | 0.92 |
| Low-density lipoprotein cholesterol, mg/dL (n = 314) | -0.018 | 0.75 | -0.049 | 0.39 |
| ***Statin therapy：No (n = 717)*** | | | | |
| Total cholesterol, mg/dL | 0.094 | 0.012 | 0.063 | 0.091 |
| High-density lipoprotein cholesterol, mg/dL | 0.014 | 0.71 | 0.026 | 0.50 |
| Non-high-density lipoprotein cholesterol, mg/dL | 0.079 | 0.033 | 0.040 | 0.28 |
| Low-density lipoprotein cholesterol, mg/dL (n = 328) | 0.067 | 0.23 | 0.033 | 0.56 |


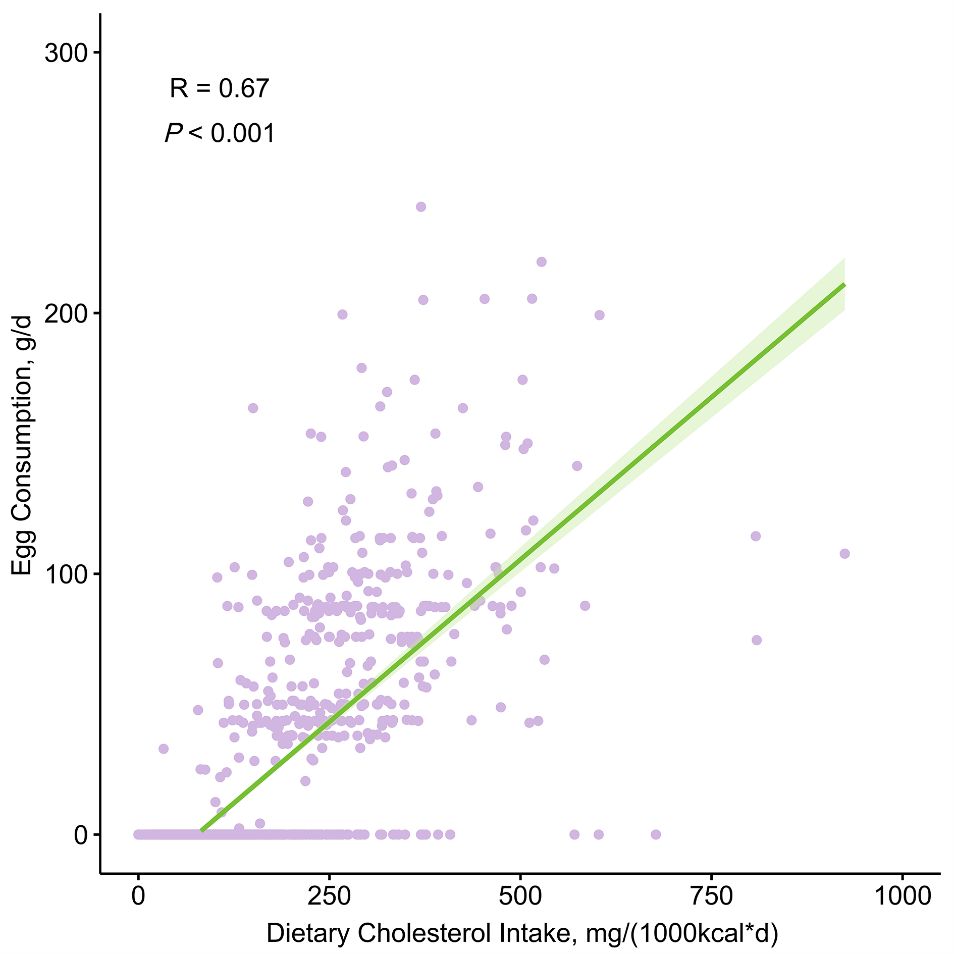


**Figure S1.** Spearman correlation coefficients between dietary cholesterol intake and egg consumption


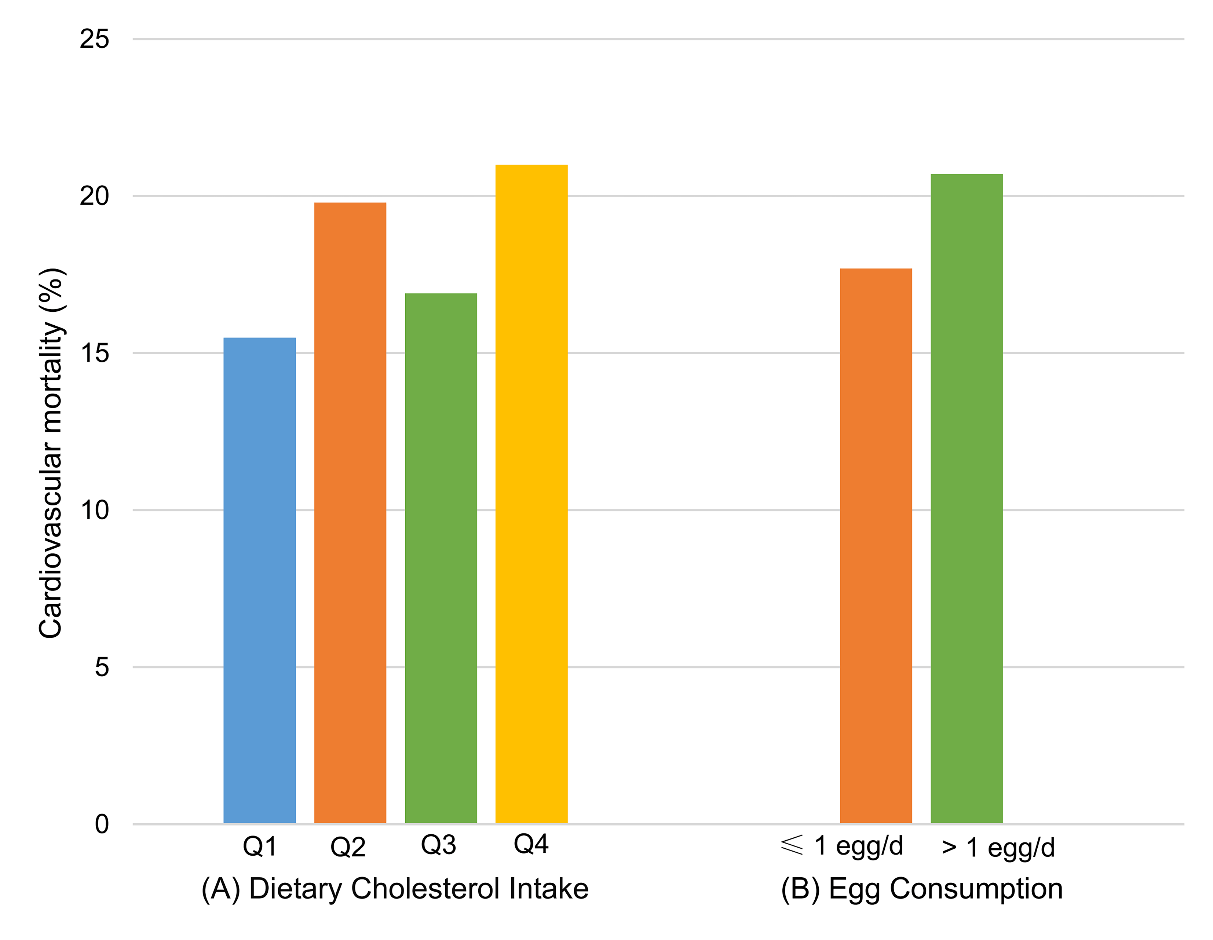


**Figure S2.** The prevalence of cardiovascular mortality across different intake groups of (A) dietary cholesterol and (B) eggs.


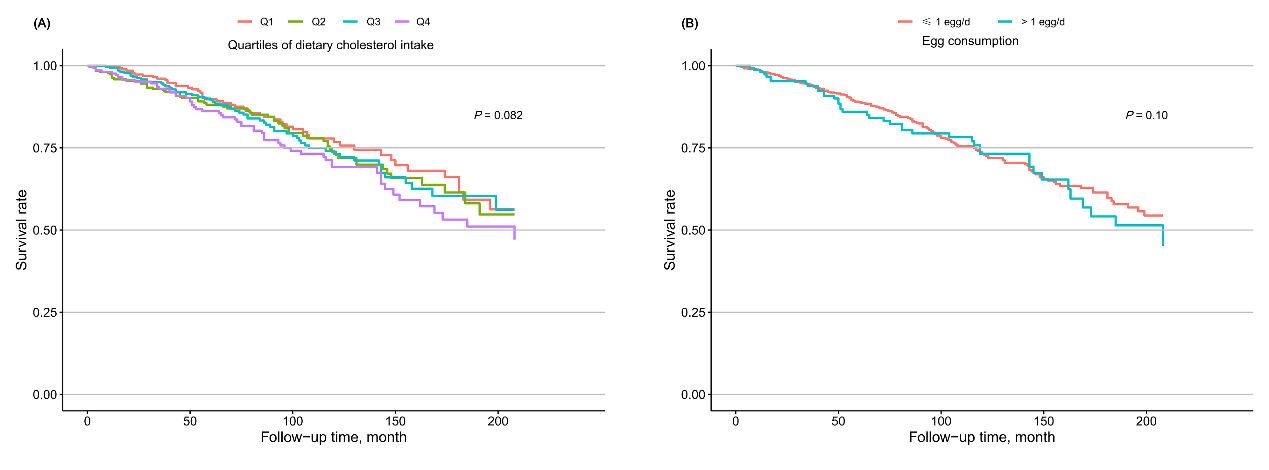


**Figure S3.** The Kaplan–Meier curve of cardiovascular mortality for the study participants with different (A) dietary cholesterol intake and (B) egg consumption


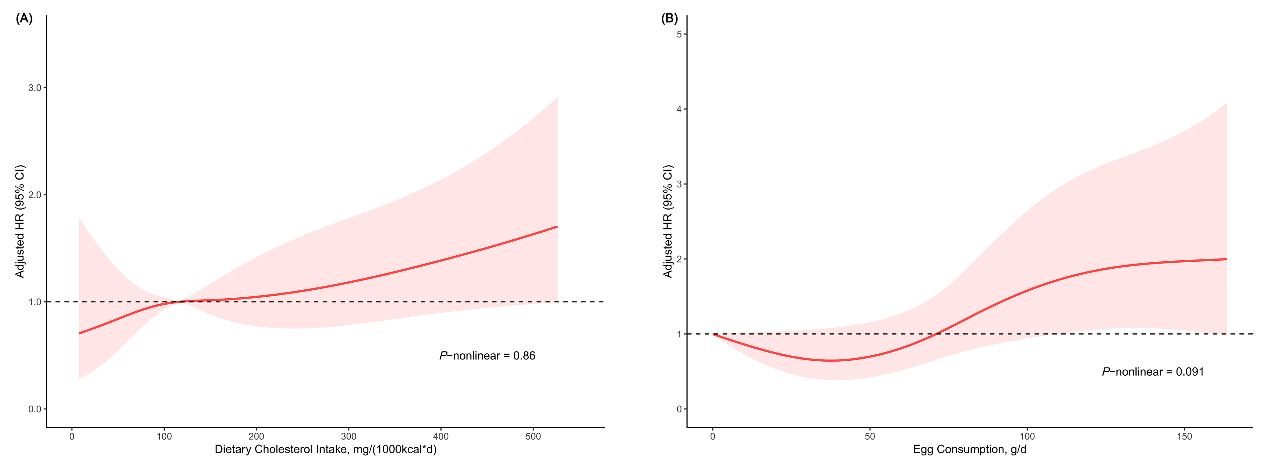


**Figure S4**. Restricted cubic spline regression model of the associations of (A) dietary cholesterol intake and (B) egg consumption with risk of cardiovascular mortality after stroke.


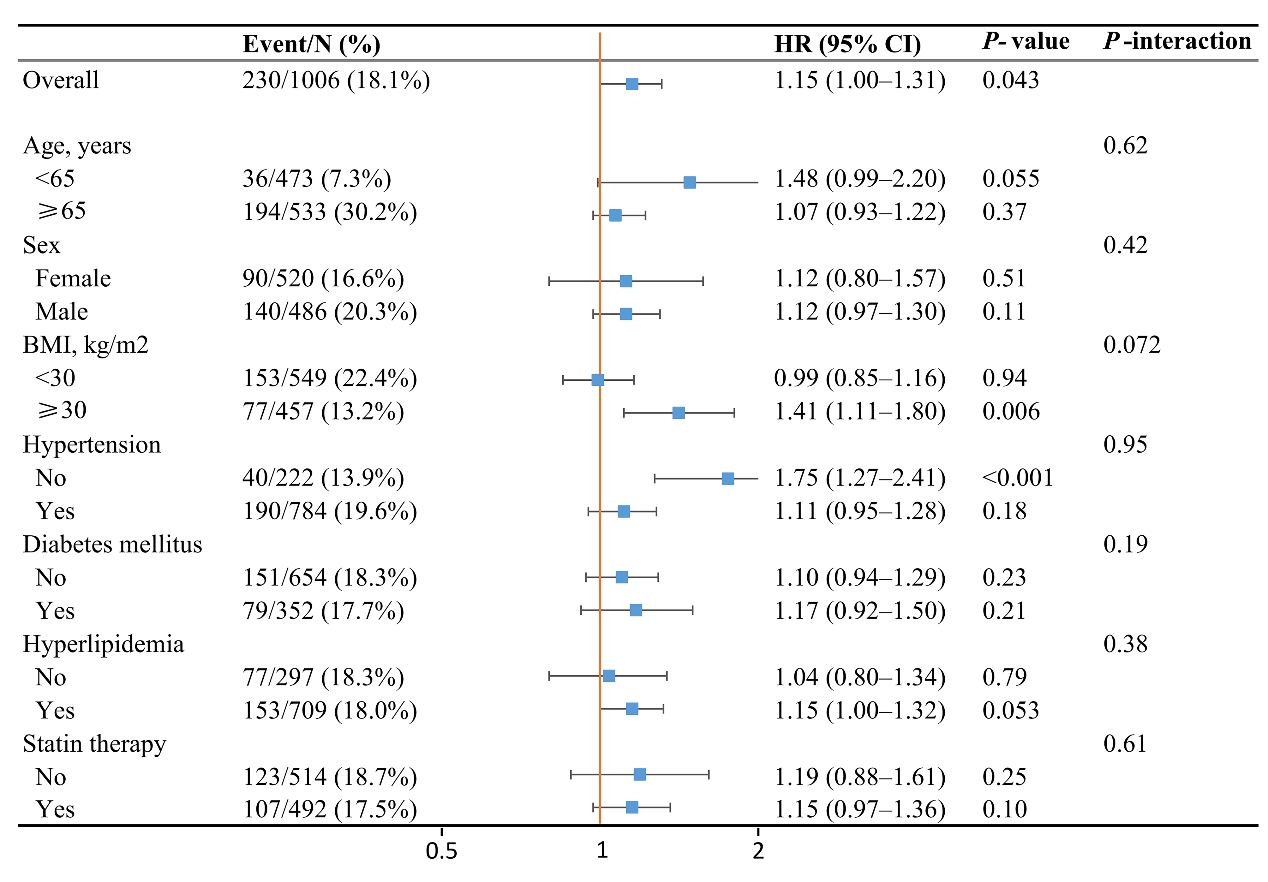


**Figure S5.** Association between dietary cholesterol intake and risk of cardiovascular mortality among different subgroups.


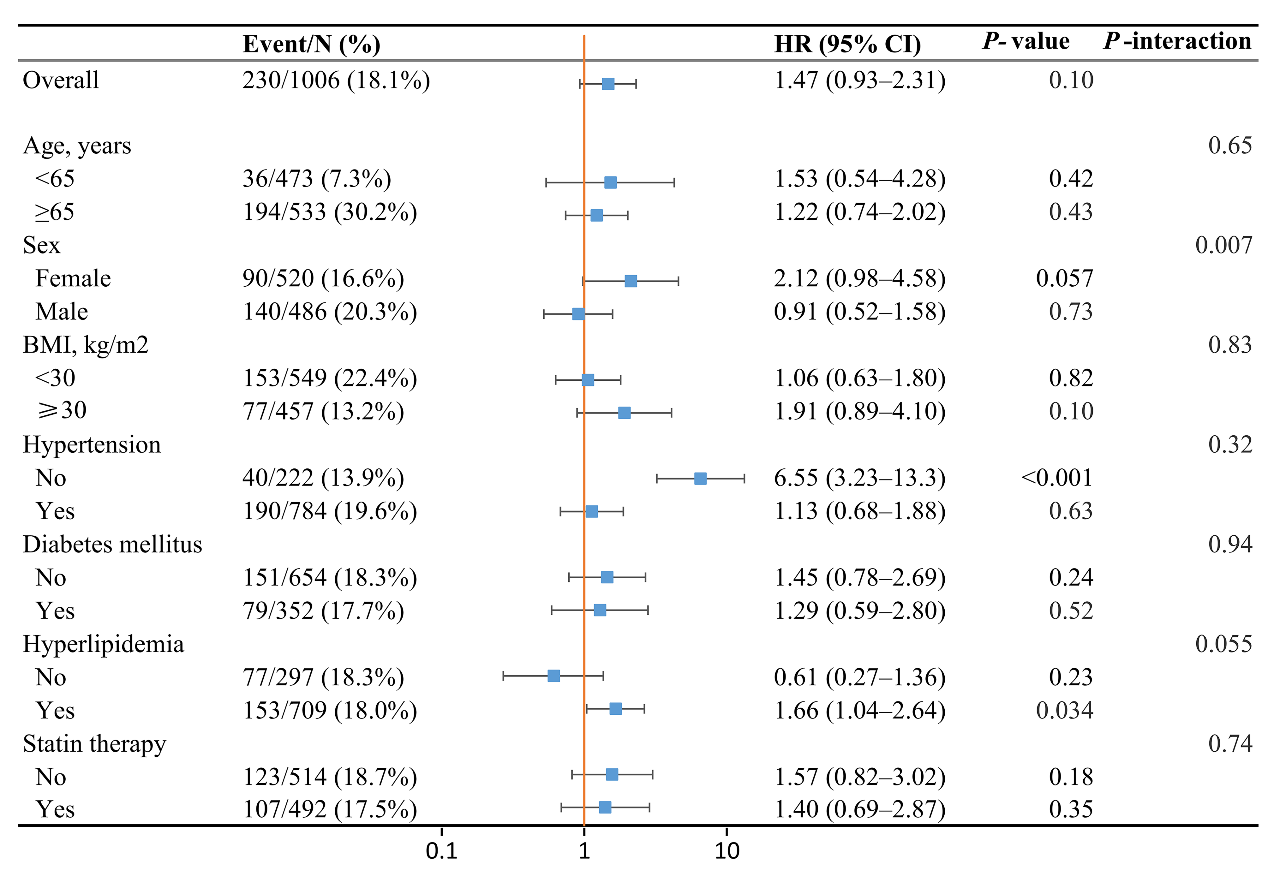


**Figure S6.** Association between egg consumption and risk of cardiovascular mortality among different subgroups.
